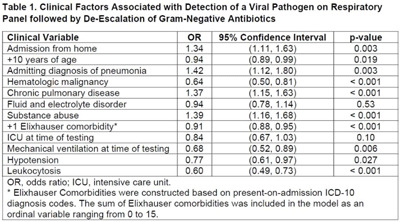# Clinical factors associated with antibiotic de-escalation after a positive multiplex molecular respiratory panel

**DOI:** 10.1017/ash.2022.218

**Published:** 2022-05-16

**Authors:** Jonathan Baghdadi, Daniel Morgan, Anthony Harris, Katherine Goodman

## Abstract

**Background:** Under ideal circumstances, multiplex molecular respiratory panels can support early all discontinuation of unnecessary antibiotics by facilitating diagnosis of viral infection. Our goal was to identify clinic situations in which a positive respiratory panel was associated with antibiotic de-escalation. We focused on gram-negative antibiotics in recognition of the urgent threat posed by gram-negative resistance. **Methods:** The sample included hospitalized adults tested by respiratory panel while receiving gram-negative antibiotics at the University of Maryland Medical Center from 2015 to 2020. Only the first respiratory panel performed during hospitalization was included. The primary outcome was the combination of a positive result on respiratory panel indicating detection of a viral pathogen and de-escalation of gram-negative antibiotics. De-escalation was assessed based on antibiotics administered on day 3 after testing and was defined by discontinuation or switch to an agent with a narrower spectrum of activity. Least absolute shrinkage and selection operator (LASSO) regression was used to construct the multivariable logistic regression model. Classification and regression tree (CART) analysis was used to identify subgroups with a higher likelihood of the primary outcome. **Results:** Of 8,326 patients, 1,462 (17.6%) tested positive by respiratory panel. The most common pathogen was rhinovirus (7.9% of the sample). Gram-negative–targeted antibiotics were de-escalated in 4,456 cases (53.5% of the sample), including 887 patients with a positive result on respiratory panel indicating a viral pathogen (60.7% of patients with a positive viral result). LASSO regression was used to select 12 variables (Table 1). Admitting diagnosis of pneumonia (OR, 1.42), comorbid substance abuse (OR, 1.39), chronic pulmonary disease (OR, 1.39), and admission from home (OR, 1.34) were associated with antibiotic de-escalation in conjunction with a positive respiratory panel. Leukocytosis (OR, 0.59), hematologic malignancy (OR, 0.64), mechanical ventilation at time of testing (OR, 0.68), and hypotension (OR, 0.77) were associated with decreased likelihood of antibiotic de-escalation in conjunction with a positive respiratory panel. CART analysis identified patients tested within 40 hours of admission as having a higher likelihood of a positive result in conjunction with antibiotic de-escalation. Among patients tested within 40 hours of admission, the probability of a positive result followed by antibiotic de-escalation was 11.9% (95% CI, 11.1%–12.8%). For patients tested >40 hours after admission, the probability was 6.0% (95% CI, 4.8%–7.2%). **Conclusions**: Targeted use of respiratory panel testing may increase the likelihood of an informative result that can drive decision making related to antibiotic use. Our exploratory analysis suggests that respiratory panel testing in the first 2 days

**Funding:** None

**Disclosures:** None

**Table:**